# Supplementary material for: Patterns of Intron Gain and Loss in Fungi
Source: PLoS Biol. 2004 Nov 30;2(12):e422. doi: 10.1371/journal.pbio.0020422 (PMC532390; doi:10.1371/journal.pbio.0020422)
Supplement: Table S1 — Also available at http://genes.mit.edu/NielsenEtAl/. (4.3 MB ZIP). [file pbio.0020422.st001.zip › NielsenEtAl/html/990.html]

AN8057.1.NCU06452.1.MG06466.1.FG07287.1


```
 CLUSTAL W (1.82) Multiple Sequence Alignments - Introns Inserted


Sequence 1: MG06466.1	375 aa
Sequence 2: FG07287.1	371 aa
Sequence 3: NCU06452.1	376 aa
Sequence 4: AN8057.1	370 aa
Alignment Length: 376 aa
Number Identitical Residues: 287 aa
Alignment Score (without introns) 11833


MG06466.1 	MFRHGIRRFTTSSIR-AAVAAAAEPTQHTINVSRAQGVAKGLTGA1IGNTPLIRLNKLSE
NCU06452.1	MFRHGVRTFATTSLRRMAAVAPQEPSQYLLNVSKAQGIAKGLTGA1IGNTPLIRLNRLSE
FG07287.1 	MFRPTARRLAAS-----AAKAAAAPTSHTLNVARAQGVSRGLTGA1IGNTPLIRLNRLSD
AN8057.1  	MFRQSVRRFATA------ALRSAAESPYNVRVSQAQGFVNGLTEA1IGNTPLIRLKRLSE
          	***   * ::::      .  .   : : :.*::***. .*** * *********::**:

MG06466.1 	ETGCEILGKAEFMNPGGSVKDRAALYVVKDAEERGLLRPGGTVVEGTAGNTGIGLAHVCR
NCU06452.1	ETGCEILGKAEFMNPGGSVKDRAALYVVKDAEERGLLRPGGTVVEGTAGNTGIGLAHVCR
FG07287.1 	ETGCEVLGKAEFMNPGGSVKDRAALYVVKDAEERGLLKPGGTVVEGTAGNTGIGLAHVCR
AN8057.1  	ETGSNILAKAEFQNPGGSVKDRAALYVVKDAEERGLLKPGGTVVEGTAGNTGIGLAHVCR
          	***.::*.**** ************************:**********************

MG06466.1 	SKGYKLVIYMPNTQSQGKIDLLRLLGAEVYPVPAVAFDNPQNYNHQARRHAERLDNAVWT
NCU06452.1	SKGYKLVIYMPNTQSQGKIDLLRLLGAEVYPVPAVAFENPENYNHQARRHAERLDNAVWT
FG07287.1 	SRGYKLVIYMPNTQSQGKIDLLRLLGAEVYPVPAVAFDNPENYNHQARRHAERLDNAVWT
AN8057.1  	SKGYKLVIYMPNTQSQGKIDLLRLLGAEVYPVPAVAFDNPENYNHKARRHAESLDNAVWT
          	*:***********************************:**:****:****** *******

MG06466.1 	NQFDNTANRRAHIETTGPEIWAQTDGKVDAFTCATGTAGTLAGVTRFLKDASGGRVKSFL
NCU06452.1	NQFDNIANRRAHIETTGPEIWAQTGGKVDAFTCATGTGGTFAGTTRYLKEVSGGRVKAFL
FG07287.1 	NQFDNTANRRAHIETTGPEIWAQTDGKVDAFTCATGTAGTLAGITRYLKDVSNGRVKSFL
AN8057.1  	NQFDNTANRRAHIETTGPEIWAQTGGKLDAFTCSTGTGGTLAGITYYLKQASGGRVKSFL
          	***** ******************.**:*****:***.**:** * :**:.*.****:**

MG06466.1 	ADPPGSVLHSYIVSGGKLIERTGSSITEGIGQGRITDNLQPDLALLDGALHISDEKSIEM
NCU06452.1	ADPPGSVLHSYFSSGGKLIERSGSSITEGIGQGRITDNLKQDVDLVDGSMTISDEKTIEM
FG07287.1 	ADPPGSVLHSYVSSGGKLNERTGSSITEGIGQGRITDNLQPDIGLVDGSLHISDEKSIKM
AN8057.1  	ADPPGSVLHSYIQSGGKLVERSGSSITEGIGQGRITDNLQPDVGTLDGSLNISDEKTIEM
          	***********. ***** **:*****************: *:  :**:: *****:*:*

MG06466.1 	VYRCLDEEGLYLGASSSLNVVAAKEVAEKLGKGHTVVTMLCDGAYRYADRLFSRKWLEDK
NCU06452.1	VYRCLDEEGLYLGASSTLNVVAAKEVAEKLGKGSTVVTMLCDGAYRYADRLFSRKWLEQK
FG07287.1 	VYRCLDEEGLYLGASSSLNVVAAKEVAEKLGKGHTVVTVLCDGAYRYADRLFSRKWLTDK
AN8057.1  	IYRCLDEEGLYLGASSALNVVAAKEVAEKLGKGSTVVTILADGAYRYADRLFSKSWLESK
          	:***************:**************** ****:*.************:.** .*

MG06466.1 	KLLSAIPKHLEKYIVLP
NCU06452.1	NLLGAIPEHLQKYIVLP
FG07287.1 	KLLGAIPKHLEKYIVLP
AN8057.1  	GLRNAIPKHLEKYIVLP
          	 * .***:**:******
```
